# Behavioral and Self-Report Cognitive Impairment Differences between Fast-Acting, Standard, and Placebo Cannabis Edibles

**DOI:** 10.21203/rs.3.rs-9621757/v1

**Published:** 2026-05-18

**Authors:** Samuel M. DiCecco, Emma Smith, Kira Sturgess, Mohammad Habib, Hollis C. Karoly, Charles Villanueva, Michael Hennesy, Bradley T. Conner

**Affiliations:** University of Minnesota; Colorado State University; William & Mary; Colorado State University; University of Colorado Anschutz Medical Campus; Azuca, Inc.; Wana Brands, LLC.; Colorado State University

**Keywords:** Blood cannabinoids, microencapsulation, acute administration, cannabis edibles, pharmacodynamics, impairment

## Abstract

**Rationale:**

Fast-acting edible cannabis products are marketed as producing quicker effects than standard edibles, but whether faster absorption translates into earlier cognitive impairment due to cannabis intoxication is unclear.

**Objectives:**

We compared fast-acting, standard, and placebo edibles in a double-blind, within-subject crossover study of 20 adult individuals who frequently use cannabis.

**Methods:**

Participants completed three mobile laboratory sessions after ingesting either a fast-acting, standard, or placebo cannabis edible and were assessed repeatedly over 270 minutes using the DRUID impairment app and selected self-report items from the Subjective High Assessment Scale (SHAS).

**Results:**

Mixed-effects models showed limited evidence that the fast-acting edible produced significantly earlier impairment than the standard edible. Behavioral impairment showed no clear pattern over time. Self-reported effects were more consistent across both active edibles, with elevations in clumsiness, distorted time perception, and other subjective impairment outcomes relative to placebo. However, the fast-acting and standard edibles generally showed similar time courses, with little evidence of a meaningful advantage for the fast-acting formulation.

**Conclusions:**

Both active edibles produced several hours of subjective impairment, but the fast-acting product did not consistently yield significantly earlier cognitive effects than the standard edible.

## Introduction

As cannabis use continues to grow in the U.S., methods of consumption are evolving and expanding to match the broader variety of products available (Baldwin et al., 2024). Edibles are the second most used cannabis product in legal U.S. markets after flower products (Goodman et al., 2020; Hammond et al., 2022; National Academies of Sciences et al., 2024). This increase in edible consumption following legalization may be a result of greater product variety, improved marketing, and reduced pulmonary risk compared to smoking (Barrus et al., 2016; Giombi et al., 2018). Edible cannabis is considered a lower-risk form of cannabis consumption, due to not relying on combustion and inhalation which is known to cause lung damage (Fischer et al., 2017). This is backed up by consumer perception studies, finding this perceived lower risk more appealing when considering product options and methods of ingestion (Choi et al., 2024; McMahon et al., 2023). However, differences in the method of administration produce distinct pharmacokinetic and pharmacodynamic profiles, leading to different experiences during the substance’s intoxicating effects, including a differing time course of intoxication, and potential differences in physiological effects and impairment (Baldwin et al., 2024; Cavaleri et al., 2024; Lankenau et al., 2018; Stella et al., 2021). In particular, orally ingested cannabis undergoes first-pass hepatic metabolism, resulting in the formation of the active metabolite 11-hydroxy-THC (11-OH-THC), which may contribute to differences in onset, intensity, and duration of intoxication compared to inhaled cannabis (Peng & Shahidi, 2021).

THC preparation and dosage (route of administration, cannabinoid formulation, etc.) can impact the onset and intensity of intoxication and impairment (Baldwin et al., 2024; Lankenau et al., 2018). Onset, duration, and intensity are further affected by individual differences, such as tolerance, sensitivity, and diet (Bloomfield et al., 2019; Cavaleri et al., 2024). While edibles may present as an easily quantifiable method of cannabis administration due to lack of variability from user preparation, intake, and specific formulations, they also have a higher incidence of adverse effects from overconsumption (Allaf et al., 2023; Monte et al., 2019; Noble et al., 2019). The delay in onset of intoxication with edible cannabis is a risk factor for overconsumption, as individuals may choose to ingest a greater amount of cannabis if they feel they have not reached the desired effect quickly enough (Schlienz et al., 2020).

Beyond the effects of acute overconsumption leading to adverse effects, route of cannabis administration also appears to affect the trajectory of the cannabis experience and effect on cognition during typical cannabis intoxication (Cavaleri et al., 2024; Curran et al., 2016). Acute administration of cannabis creates impairment in cognitive function (Bidwell et al., 2020; Bourque & Potvin, 2021). In particular, verbal learning and memory (e.g., encoding, consolidation, retrieval and working memory) are frequently identified as the cognitive domains that are most impacted by acute cannabis intoxication (Bourque & Potvin, 2021; Curran et al., 2016). The extent and time course of these effects differ depending on route of administration, as edible products lead to less detrimental effects in speed of processing compared to flower (Bourque & Potvin, 2021; Cavaleri et al., 2024). Other cognitive domains such as psychomotor functioning, attention, and executive functioning, are also thought to be negatively impacted by cannabis, but effects are less consistent (Broyd et al., 2016; Cavaleri et al., 2024). Some specific effects of cannabis intoxication, such as internal time-distortion effects, are well documented but are moderated by other variables, such as tolerance or frequency of use (Broyd et al., 2016; Sewell et al., 2013) and may also be moderated by method of preparation and administration of cannabis. It is therefore prudent to further investigate differences which exist across methods, both in intoxication and impairment effects, while attempting to account for confounding variables.

Our previous work investigated differences in pharmacokinetic properties across “fast-acting” and standard edible products. Fast-acting compounds are a growing marketed product in the legal cannabis industry (Atsmon et al., 2018; Bar-Hai et al., 2022; Izgelov et al., 2020). In some cases, these compounds do have different metabolic implications during intoxication. Microencapsulation technology used in these products encloses small particles of active substances with protective coatings, enhancing solubility and better regulating compound release. While research is still limited on the efficacy of these claims, we concluded there was an earlier onset of maximal concentration of blood THC (T_max_) in the fast-acting edible product, but the area under the curve (AUC) indicated total intoxication from the product was roughly identical (Conner et al., 2026). This finding thus implies earlier onset based on blood concentration; however, current understandings of the pharmacodynamic effects on cognitive impairment, intoxication, and physiological differences are limited.

We sought to further investigate differences between fast-acting and standard products in pharmacodynamic domains. The present study seeks to systematically compare cognitive impairment, measured both behaviorally through an impairment detection app, and through self-report measures, in fast-acting, standard, and placebo edible products. This was accomplished through a 3-way counterbalanced crossover observational study. We hypothesized that impairment measured by the impairment app and self-reported impairment would have significantly earlier onset in the fast-acting product, though both would have similar AUC measures indicating similar total impairment across the active products.

## Methods

### Participants

687 individuals were screened for eligibility through social media and public advertisements targeting the western region of the United States. Inclusion criteria required participants to be 21–65 years old, report cannabis use at least twice per week for the past 3 months, have used a cannabis edible within the past year, and report cannabis use within the past 60 days. Exclusion criteria included use of medications known to interfere with cannabis metabolism, history of serious medical or cardiovascular conditions, prior psychiatric diagnosis, or history of self-harm or suicidal ideation. Of those screened, 106 met initial eligibility criteria, whereas 465 were determined to be ineligible. An additional 49 individuals required follow-up contact to clarify eligibility status, and 7 eligible participants withdrew prior to study participation.

In total, 20 participants completed all three mobile administration sessions. Among these participants, 53% reported their sex assigned at birth as male and 47% as female. Participants ranged in age from 21 to 57 years (*n* = 20; *M* = 29.63, *SD* = 8.19). Sample characteristics are presented in Table 1. More detailed characteristics can be found as Supplementary Material 1. This study had institutional review board (IRB) approval from the Colorado State University IRB and proceeded in accordance with the ethical standards following the 1964 Declaration of Helsinki. All participants completed informed consent prior to participating in this study.

### Procedures

Participants completed three mobile laboratory sessions conducted near the participant’s residence using a mobile research unit. Sessions were separated by a minimum of four days to minimize residual drug effects and tolerance. Participants were instructed to abstain from cannabis use for at least four days prior to each session. Participants were also instructed to fast for at least eight hours before arrival. Abstinence and fasting verification for each participant was self-reported. Prior to each session, participants were provided with a standardized snack (~ 400 kcal) to reduce variability in absorption in a fed versus a fasted state.

All edible products used in the study were manufactured with identical ingredients apart from the THC formulation for each condition, including flavoring. This included a standard, fast-acting and placebo edible. Both the standard and fast-acting edible contained 10 mg of THC distillate; however, the fast-acting edible contained micro-encapsulated distillate to increase bioavailability and produce a faster onset of effects (Conner et al., 2026). The placebo edible had a negligible quantity of THC as cannabis derived terpenes were used to maintain consistent flavor of edibles across conditions (< 0.1% THC in the placebo). The study used a within-subjects, counterbalanced design in which each participant completed all three conditions in a randomized order. All conditions were double-blind. Edible products were prepared, packaged in identical food-safe containers, and labeled with condition codes by the study sponsor. Conditions, thus, were unknown to participants and study staff during testing. Participants obtained study products from a local dispensary at a discounted price prior to the first session and removed outer packaging before testing to maintain blinding.

At the beginning of each mobile laboratory session, participants reviewed and signed a second informed consent document specific to the in-person procedures. Eligibility was confirmed at each visit through verification of age (≥ 21 years), self-report of cannabis abstinence since the previous session, and biological screening. Participants completed a breath alcohol test (required BAC = 0.00), a urine drug screen for non-cannabis substances (amphetamines, benzodiazepines, cocaine, methamphetamine, and opioids), and, for participants assigned female at birth, a urine pregnancy test. Sessions were rescheduled if participants did not report successfully abstaining from cannabis or failing either the breath alcohol test or urine drug screen. Sessions were discontinued if exclusion criteria were met, following a positive pregnancy test, or had repeated failed drug and alcohol screening or cannabis abstinence.

DRUID behavioral impairment baseline, and self-report assessments were collected prior to cannabis administration. Participants were then instructed to ingest the assigned edible cannabis product at their residence while being observed remotely via Zoom to verify product administration.

After ingestion, participants returned to the mobile laboratory for repeated assessment over a 240-minute monitoring period. Self-reports of intoxication and blood samples were collected at 5, 10, 15, 30, 60, 90, 120, 150, 180, 210, 240, and 270 minutes post-ingestion. Blood sample collection and analysis procedures concurrent with the study is described in greater detail elsewhere (Conner et al., 2026). DRUID behavioral impairment was measured at all time points but the 5- and 10-minute assessments. Participants remained under continuous supervision by research staff for the duration of each session. Participants were released approximately five hours after ingestion and were instructed not to drive or operate machinery until any acute drug effects had resolved. Participants were also reminded to abstain from cannabis use until the next session.

### Measures

#### Subjective High Assessment.

To assess subjective intoxication, participants completed the Subjective High Assessment Scale (SHAS; Schuckit & Gold, 1988), a self-report measure commonly used in human laboratory studies of psychoactive substances. The SHAS consists of 15 Likert-type items assessing perceived drug strength and subjective intoxication, including feelings such as “high,” “dizzy,” and “best I have ever felt.” Participants rate each item on a continuous scale indicating the intensity of the effect at the current moment, with higher scores reflecting greater subjective intoxication. The measure has been widely used in alcohol and nicotine studies and has shown sensitivity to dose-dependent subjective effects (Ray et al., 2012; Schuckit & Gold, 1988). For the purposes of the current study in focusing on domains of cognitive impairment, particular attention was given to the following questions: “Clumsy” (SHAS3), “Confused” (SHAS4), “Slurring of Speech” (SHAS5), “Dizzy” (SHAS6), having a “Distorted Sense of Time” (SHAS10), “Difficulty Concentrating” (SHAS12), and “Feelings of Floating” (SHAS13). These subjective reports on experience from the SHAS adequately capture cognitive impairment domains affected by cannabis, particularly psychomotor functioning, working memory, executive functioning, attention, and time dilation.

#### DRUID.

Behavioral impairment was quantified by performance on the DRUID mobile app, an iPad application meant to quantify psychomotor and cognitive impairment. The app consists of four 30–45-second tasks, assessing reaction time, decision making, motion tracking, and time estimation under conditions of divided attention, as well as a separate balance task (Richman and May, 2019). These domains are considered reliable predictors of acute substance-related impairment, including cannabis impairment (Mikulskaya & Martin, 2018). DRUID then provides a “global impairment score” via a proprietary algorithm using individual task measurements. This value is meant to indicate, relative to an individual’s precalculated baseline, whether they are cognitively impaired and to what degree. This global impairment score acts as this study’s outcome variable of behavioral impairment, ranging from 0–100.

### Data Analysis

Participants’ data were recorded at thirteen time points in one session for each edible, creating a three-level crossed design with level one being the timepoint, level two being the edible type, and level three being the individual. Outcome variables included the behavioral impairment (DRUID) score, and the SHAS items listed previously. All DRUID scores were normally distributed. All SHAS variables were count distributed and zero-inflated. Predictors included time, edible type (standard, fast-acting, or placebo) and the interaction between time and edible. Time was included as a quadratic predictor for all models, except for Slurring of Speech where it was only included as a linear predictor due to no quadratic relationship. All models included an autoregressive factor (ar1), except for Confusion and Dizziness as those variables lacked enough variance to include an autoregressive factor. All models included a random intercept for edible type and a random intercept for each individual. All models included age, assigned sex, past 30-day cannabis use frequency (as a proxy measure of tolerance), and body mass index (BMI; to account for height and weight), as covariates.

To interpret the model results, we calculated average marginal effects (AMEs) (Mize, 2019). Through calculating AMEs we investigated the effect of edible type on the outcomes averaged across all timepoints and the effect of time on the outcomes averaged across all edible types. Due to the anticipated quadratic form following intoxication and then descent, we then conducted pairwise comparisons of the AMEs of time on the outcomes using second differences, to identify differences based on the edible products. Lastly, we examined the conditional marginal effect of time for each edible at each time point post consumption to examine the onset, peak, and offset of the impairment effect for each product.

To further probe the interaction through estimated values at each time point, we calculated estimated marginal means, producing estimated counts/probabilities for the outcome for each edible at every timepoint. We then compared the estimated marginal means using pairwise comparisons and adjusting the p-value for multiple comparisons using the Benjamini-Hochberg procedure (Benjamini & Hochberg, 1995) in order to decrease the likelihood of a Type 1 error.

## Results

**Figure 1:**
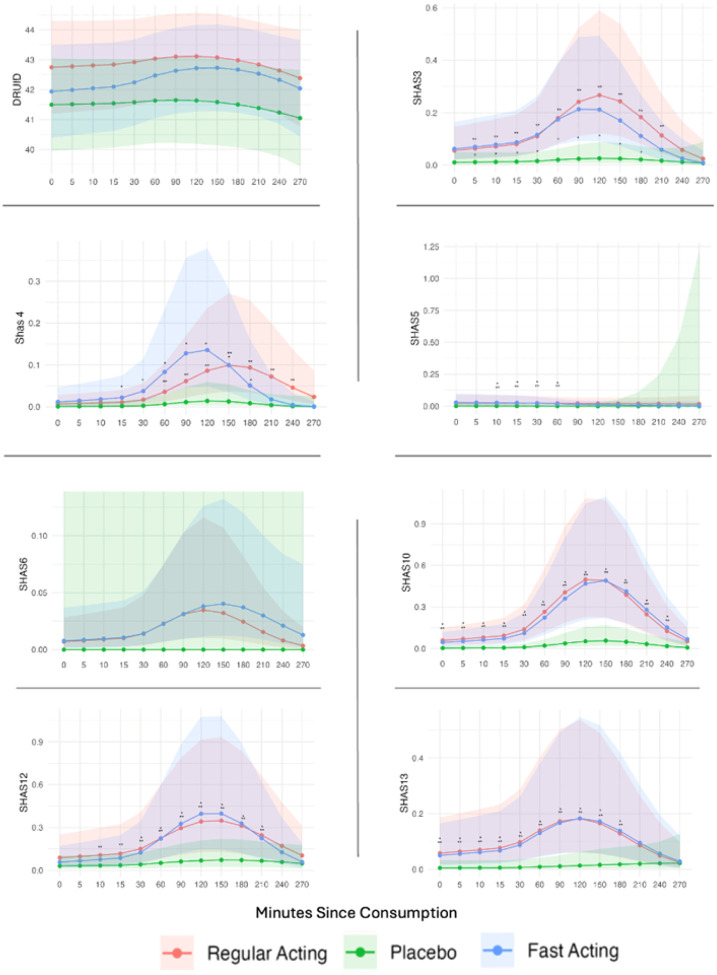
Model and estimated marginal means of impairment trajectory across cognitive impairment variables. Y axis indicates predicted outcome variable generated at mean values for age, sex, past 30 day use, and bmi. * indicates difference in outcome variable estimated marginal mean at that time point between placebo and fast-acting edible. ** indicates significant difference in outcome variable estimated marginal mean between placebo and standard edible.

The first difference AMEs of covariates and paired differences between edibles for each of the eight outcome variables is presented in Table 2. Complete information on the first differences at each time point is presented in Supplementary Material 2, and pairwise comparisons in conditional slope at each time point are presented in Supplementary Material 3. A summary of the beginning and end point of impairment, quantified as when the product has a significant slope difference from the placebo product, are presented here. Depictions of the overall predicted model trajectory and pairwise differences between estimated marginal means can be observed in Fig. 1.

### Estimated Mixed Modeling (Average Marginal Effects and Estimated Marginal Effects)

For DRUID, there was no overall effect of time averaged across all edibles. Averaged across all timepoints, only the standard edible differed from the placebo edible, such that the standard edible had higher DRUID scores on average across all timepoints as compared to the placebo edible (AME = 1.401, p = 0.012). Of note, there was a positive association with DRUID scores and the covariate of age, such that as age increased the average DRUID score across all edibles and times increased (AME = 2.17, p = 0.03). There was a significant conditional pairwise difference between the standard edible and placebo from 30–210 minutes, but notably there were no times at which there was a significant difference for the fast-acting edible from the placebo, seeming to indicate an inability to detect impairment. There were no differences in estimated marginal means at any of the timepoints between any edibles.

For Clumsiness, there was an overall effect of the time such that as time increased Clumsiness also increased, averaged across all edibles (AME = 0.0002, p = 0.01). There was a positive association at 30-minutes (AME = 0.0016, p = 0.013), no association at 120-minutes (AME = −0.00024, p = 0.45) and a negative association at 210 minutes post consumption (AME = −0.0014, p = 0.013), exemplifying a quadratic effect. Averaged across all timepoints, both the fast-acting (AME = 0.101, p = 0.022) and standard edibles (AME = 0.127, p = 0.015) had greater Clumsiness scores than the placebo. The fast-acting edible had a greater estimated marginal mean compared to the placebo edible from 5–180 minutes. The standard edible had a larger estimated marginal mean from 5–210 minutes post consumption. Conditional slopes in the estimated model were significantly different from the placebo edible from 30–180 minutes in the fast-acting edible, and from 30–210 minutes in the standard edible. Together, this indicates a longer time experiencing clumsiness in the standard edible condition, but not a significantly earlier starting time or earlier magnitude increase in the fast-acting condition.

In Confusion, there was no effect of time on Confusion averaged between any edible, nor any differences between edible types averaged across all timepoints. The estimated marginal means for the fast-acting edibles were significantly greater than the placebo edible from 15 to 180 minutes post consumption. The estimated marginal means for the standard edible were greater than the placebo edible from 60 to 240 minutes post consumption, seeming to indicate a lagged start time but similar curve. The conditional slopes in the estimated model however were not significantly different from placebo at any individual point for either fast-acting or placebo.

There was no effect of edible type or time on Slurring of Speech averaged across all edibles. Averaged across all timepoints, the standard edible had greater Slurring of Speech scores than the placebo edible (AME = 0.069, p = 0.039). The estimated marginal means for both the fast-acting and standard acting edibles were greater than the means for the placebo edible from 10 to 60 minutes post consumption, while the conditional slopes were only significantly different from placebo for the standard edible from 60 minutes to 150 minutes.

For Dizziness, averaged across all edibles, there was a positive effect of time on Dizziness (AME = 0.0001, p = 0.023). Across all timepoints, there was an effect of time on Dizziness for both the fast (AME = 0.0002, p = 0.04) and standard edibles (AME = 0.0001, p = 0.041). However, there were no significant different pairwise differences from placebo. There were no differences in estimated marginal means or conditional slopes at any time point between any edibles.

Averaged across all edible types, there was a positive effect of time on Distortion of Time (AME = 0.001, p = 0.005). There was a positive effect averaged across all edibles at 30 minutes post consumption (AME = 0.003, p = 0.006) and at 120 minutes post consumption (AME = 0.002, p = 0.03). There was a negative effect of time averaged across all edibles at 210 minutes post consumption (AME = −0.0051, p = 0.008), indicating a quadratic effect. Averaged across all timepoints, both the fast (AME = 0.31, p = 0.021) and standard edibles (AME = 0.32, p = 0.016) had greater Distortion of Time values than the placebo edible. There was a positive effect of time on Distortion of Time for both the fast-acting (AME = 0.0007, p = 0.015) and standard edibles (AME = 0.0008,, p = 0.012). The estimated marginal means for both the fast-acting and standard edibles were greater than the placebo edible from 0 minutes until 240 minutes post consumption, indicating a similar time course. The conditional slopes compared to placebo were similar, with the standard edible being slightly earlier at 5 minutes in the estimated model.

For Difficulty Concentrating, when averaged across all edible types there was no effect of time on Difficulty Concentrating and averaged across all timepoints, there were no differences in Difficulty Concentrating for edible types. When averaged across all timepoints there was no difference in Difficulty Concentrating between any of the edible types. There was no effect of time for any of the edibles across all time points, or in any conditional slopes at individual time points. The estimated marginal means for the fast-acting edible was greater than that of the placebo edible from 30 until 210 minutes. Similarly, the estimated marginal means of the standard edible were greater than that of the placebo edible from 10 to 210 minutes, suggesting earlier onset of Difficulty Concentrating for the standard edible.

Finally, averaged across all edible types, there was a positive effect of time on Feelings of Floating (AME = 0.0002, p = 0.049). There was no effect of time across all timepoints for any of the edibles, nor between any of the edibles. There were no differences in conditional slope pairwise differences between either the fast-acting or standard edible for any time. The estimated marginal means for the fast-acting and standard edible were greater than that of the placebo edible from 0 minutes until 180 minutes post consumption, seeming to indicate similarly estimated time courses.

### Non-parametric Time Series Comparisons across edibles

**Figure 2:**
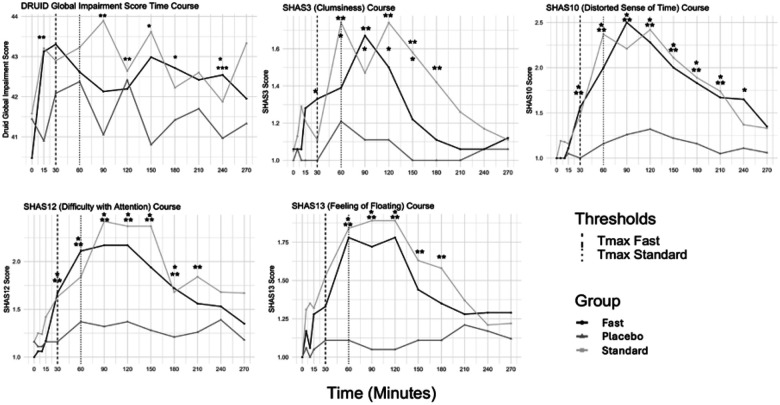
Plot of the raw means in each outcome variable over Time. Thresholds indicate the T_max_ values identified by previous studies in the same edible products (Conner et al., in press), with the dashed lines indicating T_max_ for the fast-acting edible at 30 minutes, and the dotted indicating T_max_ for the standard edible at 60 minutes. * indicates difference in outcome variable estimated marginal mean at that time point between placebo and fast-acting edible. ** indicates significant difference in outcome variable estimated marginal mean between placebo and standard edible.

The estimated marginal means at each time point assess differences in the estimated mean from the identified model at each timepoint. However, due to some discrepancies still existing in the overall best fitting models, such as differences in estimated marginal means at 0 minutes for Distorted Sense of Time and Feelings of Floating, or heavily overlapping standard errors such as in the Dizziness model, it was prudent to also investigate individual comparisons at each time point to assess differences between each edible product with the raw data. Further, we sought to compare differences between the peaks of each curve as well as the differences at the associated T_max_ for each edible product using the actual data as opposed to estimated marginal means. These differences in associated T_max_ are described in greater detail in previous publications, finding that T_max_ for the standard product was at 60 minutes and 30 minutes for the fast-acting edible (Conner et al., 2026). An arbitrary value of 60 minutes for T_max_ of the placebo was also selected for comparison. Non-parametric Wilcoxon Signed Ranks Tests were used for comparisons between standard - placebo, fast-acting - placebo, and standard - fast-acting due to persistent non-normality in all variables other than DRUID behavioral impairment. Wilcoxon Tests were also used for the DRUID analysis for parsimony. Plots of the raw data between edible products are presented in Fig. 2. Full tables for the pairwise tests can be found in Supplementary Material 3.

For DRUID, the Wilcoxon tests identified several differences across the overall time course between different products, but with no clear pattern emerging. The standard edible was significantly increased from placebo at 15 minutes, 90 minutes, and 120 minutes. The fast-acting was significantly different from placebo at 150, 180 minutes, and 240 minutes. The fast-acting was also significantly different from the standard edible at 240 minutes. However, the large values of standard error throughout the time course make identifying conclusions difficult for this variable. In addition, there were no differences at T_max_ or at the peaks between each product or to the placebo.

For Clumsiness, there were significant differences between the standard product from 60–180 minutes, and 30–150 minutes in the fast-acting product. There were no differences between T_max_ times and peaks for Clumsiness between the active products; differences were observed only relative to placebo. Distortion of Time had significant differences for both active edibles from 30–240 minutes, but with the fast-acting edible lasting slightly longer to 240 minutes. Further, at its respective T_max_, the standard edible was found to be different from placebo, but the fast-acting was not. There were no differences between active product peaks for Distortion of Time, but again difference compared to placebo. For Difficulty Concentrating, impairment began at 30 minutes for both products, ending at 180 minutes for the fast-acting edible and 210 minutes for the standard edible. Further, both products had greater reported difficulty concentrating compared to placebo at their peak but not different to each other. Finally, the Feeling of Floating had significant differences for both products compared to placebo at 60 minutes to 120 minutes for the fast acting and to 180 in the standard edible, indicating a prolonged feeling of floating in the standard edible condition. At their respective T_max_, Feeling of Floating was different to placebo, but not between placebo and fast-acting. Again, peak differences only existed between the two active products with the placebo, and not to each other. The other outcome variables of interest (Confusion, Slurring of Speech, Dizziness) did not have more than two significant differences to placebo during the entire time course and are thus difficult to interpret a pattern.

## Discussion

The present study compared behavioral and self-reported cognitive impairment following administration of standard, fast-acting, and placebo edible cannabis products in a within-subjects, double-blind, crossover design. Overall, the findings provide partial support for the hypothesis that the fast-acting edible would produce an earlier onset of impairment than the standard edible. The fast acting and standard edibles showed a broadly similar total impairment trajectory. Across most outcomes, the fast-acting and standard products produced comparable effects, and in several cases the standard edible showed equal or greater evidence of impairment relative to the placebo than the fast-acting product.

DRUID global impairment score did not show a clear overall time effect, and overall did not map neatly onto a pattern of cognitive impairment. This finding is consistent with a prior study investigating the acute impairment effects of edible cannabis using DRUID, finding that 10 mg doses did not impair cognitive performance, but produced positive subjective effects (Spindle et al., 2021). Given that these findings were also explicitly in people with are less experienced with cannabis, it is not unexpected that participants in the present study did not become impaired enough for DRUID to detect impairment (Spindle et al., 2021). Previous work using the DRUID app found that average peak impairment scores following acute use of smoked cannabis were over 50 (which is in the moderate impairment range) (Karoly et al., 2022), while in the current study, peak impairment was below 44 (unimpaired range) on average for all conditions. In the study conducted by Spindle and colleagues (2021), only 20% of their sample taking the 10 mg dosage were considered impaired via DRUID. Similarly in our study, only five participants (25%) reached 50 at any point during the study, with only three participants (15%) consistently hitting this moderate impairment benchmark. It is also notable that the DRUID app provided the only example within the eight outcome variables of a covariate, age, being significantly associated with the outcome variable in the overall model (AME = 0.22, p < .05). Older participants showed higher average impairment scores across conditions, which may reflect age-related differences in psychomotor performance, cannabis sensitivity, or baseline test performance rather than a true age-by-product interaction, at least in our current study. This along with the lack of a significant difference between either active edible and placebo, indicates a possibility that in the current study, much of the variance being accounted for in the model may be more associated with age rather than impairment. However, because age was not a focal moderator in the study, this finding should be interpreted cautiously. Ultimately, it is not unexpected given prior findings that the cannabis dosage given to participants in this study was not high enough to produce significant behavioral impairment in this sample.

Self-reported impairment outcomes in the best fitting mixed-model showed a more consistent pattern for several of the outcomes than the behavioral impairment score, but again not one that strongly favored the fast-acting formulation. The outcome variables of Clumsiness, Confusion, Distorted Sense of Time, Difficulty Concentrating, and Feelings of Floating all had results suggesting impairment, whereas Slurring of Speech and Dizziness results were too inconsistent for interpretation. For Clumsiness, there appeared to be a longer effect found for the standard edible, while the Wilcoxon comparisons suggest that the effects of the fast-acting edible may have indeed began earlier. With Confusion, there appears to be a clear shift where the fast-acting product is delayed compared to the impairment seen in the standard product via differences between estimated marginal means, however, the best fitting model does not identify a significant difference between the active and placebo products. Distorted Sense of Time emerged as one of the clearest subjective effects of cannabis impairment, consistent with prior work showing that time distortion is a robust and common acute cannabis effect (Sewell et al., 2013). Both products however were essentially identical in the best fitting model, though the Wilcoxon comparisons indicate a longer effect in the fast-acting. Indeed, the Wilcoxon comparison of impairment at each respective T_max_ implies that the earlier T_max_ does not affect the time course of impairment with time perception. Difficulty Concentrating appeared to have an earlier onset and delayed end of impairment in the standard product, though only different from placebo in the Wilcoxon comparisons and estimated marginal means. Finally, Feelings of Floating had identical onsets in the best fitting model but appear to last significantly longer in the standard product by over 60 minutes via the Wilcoxon comparisons. Overall, Clumsiness showed the only instance of earlier onset in the fast-acting condition, while also ending earlier, implying a fully earlier lagged cycle of Clumsiness.

Prior research has shown that oral cannabis products can produce delayed and sometimes prolonged subjective effects relative to inhaled cannabis, but the exact onset and intensity can vary substantially across individuals and products (Gibson et al., 2024). In the current study, the broad overlap between fast-acting and standard products suggests that, for many impairment domains, the practical differences between these formulations may be smaller than suggested. The combination of model-based marginal effects and non-parametric time-series tests suggested that some outcomes followed broadly similar trajectories across active edibles but with modest lags between them, while others showed only sporadic significant differences at individual time points. This pattern likely reflects several factors including individual differences in tolerance, metabolism, and prior use, which are known to moderate cannabis intoxication and cognitive impairment.

Several limitations of the present study likely influenced the pattern of results. First, the sample size was small, which limited power to detect subtle differences between the active products and made the estimates vulnerable to instability across models and time points, limiting generalizability and statistical precision. Second, the use of a single 10 mg dose may have been insufficient to produce acute psychomotor impairment in our sample of people who regularly use cannabis with some degree of tolerance. This is evidenced by the lack of significant findings with behavioral impairment on the DRUID, and that the range of responses for many of the SHAS variables were relatively small.

It is important to note that the 10 mg dose was selected because it reflects the typical dosage found in consumer recreational cannabis edible products, and is actually the maximum amount of THC allowed per dose in Canada, Colorado, and other U.S. States (Donnan et al., 2024; Doran & Papadopoulos, 2019; Parnes et al., 2018). Thus, the 10 mg dose is highly ecologically valid. However, people with more cannabis experience often take several doses at once, or use multiple products at the same time (Parnes et al., 2018). Our inclusion criteria (using cannabis at least twice per week for the past 3 months) suggests that our sample includes people with more cannabis experience who may be used to taking more than 10 mg edible THC in a single sitting. We did account for variance in cannabis consumption through the inclusion of past 30-day cannabis use as a covariate, which was rather high (mean = 21.80 days, SD = 8.00). Thus, it is possible that the magnitude of impairment effects we observed were attenuated in our sample. The acute impairing effects of a 10 mg edible may be greater in a sample of individuals who infrequently use cannabis; however, safety concerns prevented us from including people with less cannabis experience in this study.

Additionally, participants completed sessions following an overnight fast and consumed a standardized snack prior to dosing. Although this controlled for variability in absorption conditions, it may not reflect typical real-world consumption, where higher fat intake can enhance oral THC absorption and alter the formation of active metabolites such as 11-hydroxy-THC. These factors could influence both pharmacokinetic profiles and downstream impairment outcomes.

These findings have practical implications for both consumers and regulators. Fast-acting edibles are marketed as producing a quicker and more controllable experience, yet the present results suggest that faster blood THC onset may not translate into measurably earlier or distinct impairment on commonly used cognitive and self-report indicators, even if they reach peak blood levels earlier. This could be reassuring from a safety standpoint, as it suggests that microencapsulation does not necessarily yield disproportionate impairment. Future work should include larger samples of individuals with regular or infrequent cannabis experience alongside direct measurement of both THC and active metabolites and impairment outcomes, as well as with additional measures of cognition. Studies should also examine whether the fast-acting formulation shows clearer advantages at different doses.

In conclusion, the present study suggests that fast-acting edible cannabis which produce earlier pharmacokinetic effects may not yield a consistent difference in behavioral or subjective impairment outcomes. The standard and fast-acting products produced broadly similar impairment trajectories, with exceptions being the standard product lasting longer in Clumsiness, Difficulty Concentrating, and Feelings of Floating, and fast-acting being earlier in Clumsiness and lasting longer in Confusion and Distortion of Time. Overall, results suggest that impairment-related risks associated with fast-acting and standard edibles are likely similar. These findings underscore the importance of distinguishing between faster absorption and faster functional impairment, and they caution against the assumption that pharmacokinetic differences will translate into meaningfully different cognitive effects.

## Supplementary Material

This is a list of supplementary files associated with this preprint. Click to download.

- CogImpairmentEdibleSupplementaryMaterial13.docx
- CogImpairmentEdibleSupplementaryMaterial4.pdf

## Figures and Tables

**Table 1:** Study Sample Descriptive Characteristics

Variable	n / %
**Age**	
Mean (years)	29.63
Range	21–57
**Sex**	
Male	53%
Female	47%
**Gender identity**	
Cisgender man	45.45%
Cisgender woman	36.36%
Gender non-conforming	9.09%
Non-binary	4.55%
Transgender woman	4.55%
**Race / Ethnicity**	
White	75.51%
Black	6.12%
American Indian / Alaska Native	5.10%
Another race	4.08%
Asian	3.06%
Native Hawaiian / Pacific Islander	2.04%
Arab or Muslim	1.02%
Prefer not to respond	3.06%

**Table 2:** Main Effects and Pairwise Contrast Comparisons Between AMEs

	DRUID			SHAS 3		
	Main Effects			Main Effects		
Predictor	AME	95%CI	p	AME	95%CI	p
Time	−0.000238	(−0.003, 0.002)	0.8533	**0.000222**	**(0.000, 0.000)**	0.0103 *
**Age**	**0.2187**	**(0.021, 0.416)**	0.0298 *	−0.002	(−0.019, 0.008)	0.684
BMI	−0.186	(−0.451, 0.080)	0.17	−0.00796	(−0.022, 0.006)	0.274
Past 30 Day Cannabis Use	0.057	(−0.110, 0.223)	0.5	−0.0052	(−0.015, 0.004)	0.286
Assigned Sex (Female - Male)	2.17	(−0.476, 4.830)	0.108	0.042	(−0.090, 0.174)	0.5329
	Pairwise Differences			Pairwise Differences		
Fast - Placebo	0.922	(−0.172, 2.017)	0.0987	**0.1009**	**(0.014, 0.188)**	0.0224 *
**Standard - Placebo**	**1.401**	**(0.307, 2.495)**	0.0121 *	**0.1273**	**(0.025, 0.230)**	0.0149 *
Fast - Standard	−0.479	(−1.574, 0.616)	0.3916	−0.0265	(−0.106, 0.053)	0.5121
	SHAS 4			SHAS 5		
	Main Effects			Main Effects		
Predictor	AME	95%CI	p	AME	95%CI	p
Time	0.000217	(0.000, 0.000)	0.0535	−0.000137	(0.000, 0.000)	0.2468
Age	−0.0018	(−0.002, 0.012)	0.7974	−0.0073	(−0.016, 0.001)	0.0866
BMI	−0.0235	(−0.058, 0.011)	0.1785	−0.0035	(−0.009, 0.002)	0.2194
Past 30 Day Cannabis Use	−0.015	(−0.037, 0.008)	0.1947	−0.00729	(−0.014, 0.000)	0.0528
Assigned Sex (Female - Male)	0.183	(−0.072, 0.438)	0.16	0.04557	(−0.022, 0.113)	0.1873
	Pairwise Differences			Pairwise Differences		
Fast - Placebo	0.143	(−0.023, 0.308)	0.0908	0.0504	(−0.006, 0.106)	0.0774
Standard - Placebo	0.13	(−0.018, 0.277)	0.0856	**0.0691**	**(0.004, 0.135)**	0.0388 *
Fast - Standard	0.013	(−0.064, 0.090)	0.7388	−0.0187	(−0.084, 0.046)	0.5739
	SHAS 6			SHAS10		
	Main Effects			Main Effects		
Predictor	AME	95%CI	p	AME	95%CI	p
Time	0.000121	(0.000, 0.000)	0.0232 *	**0.000521**	**(0.000, 0.001)**	0.00517 **
Age	−0.0204	(−0.049, 0.008)	0.1553	−0.0338	(−0.078, 0.010)	0.13276
BMI	0.000922	(−0.009, 0.011)	0.8518	−0.03113	(−0.075, 0.013)	0.16556
Past 30 Day Cannabis Use	−0.0122	(−0.028, 0.003)	0.1231	−0.007125	(−0.029, 0.015)	0.52856
Assigned Sex (Female - Male)	0.242	(−0.090, 0.573)	0.1533	−0.012967	(−0.314, 0.288)	0.93267
	Pairwise Differences			Pairwise Differences		
Fast - Placebo	0.1403	(−0.010, 0.291)	0.0677	**0.3048**	**(0.045, 0.564)**	0.0213 *
Standard - Placebo	0.1085	(−0.001, 0.218)	0.053	**0.319**	**(0.059, 0.579)**	0.0162 *
Fast - Standard	0.0318	(−0.061, 0.125)	0.5022	−0.0142	(−0.259, 0.230)	0.9092
	SHAS12			SHAS13		
	Main Effects			Main Effects		
Predictor	AME	95%CI	p	AME	95%CI	p
Time	0.00049	(0.000, 0.001)	0.051	**0.000213**	**(0.000, 0.000)**	0.0497 *
Age	−0.01666	(−0.057, 0.024)	0.4233	−0.0105	(−0.029, 0.008)	0.2692
BMI	−0.03829	(−0.105, 0.029)	0.2629	0.000643	(−0.015, 0.016)	0.9352
Past 30 Day Cannabis Use	−0.01403	(−0.048, 0.020)	0.4123	0.000297	(−0.010, 0.010)	0.9546
Assigned Sex (Female - Male)	0.13131	(−0.314, 0.576)	0.5631	0.0938	(−0.097, 0.285)	0.3356
	Pairwise Differences			Pairwise Differences		
Fast - Placebo	0.2175	(−0.047, 0.482)	0.1067	0.11613	(−0.022, 0.254)	0.099
Standard - Placebo	0.2299	(−0.042, 0.502)	0.0978	0.11823	(−0.017, −0.254)	0.0874
Fast - Standard	−0.0124	(−0.211, 0.186)	0.9027	−0.00211	(−0.140, 0.136)	0.9761
